# Delayed Effect of Craniotomy on Experimental Seizures in Rats

**DOI:** 10.1371/journal.pone.0081401

**Published:** 2013-12-04

**Authors:** Patrick A. Forcelli, David Kalikhman, Karen Gale

**Affiliations:** Department of Pharmacology and Physiology, Georgetown University, Washington, District of Columbia, United States of America; University of Pittsburgh, United States of America

## Abstract

Neurosurgical therapeutic interventions include components that are presumed to be therapeutically inert, such as craniotomy and electrode implantation. Because these procedures may themselves exert neuroactive actions, with anecdotal evidence suggesting that craniotomy and electrode placement may have a particularly significant impact on epileptic seizures, the importance of their inclusion in sham control groups has become more compelling. Here we set out to test the hypothesis that craniotomy alone is sufficient to alter experimental seizures in rats. We tested adult male rats for seizures evoked by pentylenetetrazole (70 mg/kg) between 3 and 20 days following placement of bilateral craniotomies (either 2.5 or 3.5 mm in diameter) in the parietal bone of the skull, without penetrating the dura. Control (sham-operated) animals underwent anesthesia and surgery without craniotomy. We found that craniotomy significantly decreased the severity of experimental seizures on postoperative days 3, 6, and 10; this effect was dependent on the size of craniotomy. Animals with craniotomies returned to control seizure severity by 20 days post-craniotomy. These data support the hypothesis that damage to the skull is sufficient to cause a significant alteration in seizure susceptibility over an extended postoperative period, and indicate that this damage should not be considered neurologically inert.

## Introduction

The use of surgical controls in clinical neurosurgical trials has been a topic of active debate [see [1], [2] for two opposing perspectives; and [3] for a review]. Although including controls for invasive surgical procedures in clinical trials has raised ethical questions, recent reports suggesting that procedures such as craniotomy and electrode implantation may alter brain excitability [4], [5] have strengthened the scientific argument for surgical controls.

For example, diagnostic electrode implantation alone can reduce postoperative seizure incidence in patients with epilepsy (e.g., [4], [5]). Similar findings have been obtained after implantation of electrodes for deep brain stimulation (DBS). In a small trial, Lim and colleagues found a 67% reduction in seizures in the period following implantation, before initiation of stimulation [6]. Two large-scale clinical trials of DBS in epilepsy corroborate this observation. Fisher and colleagues of the SANTE (Stimulation of the Anterior Nucleus of the Thalamus for Epilepsy) study group reported that at one month after surgery, an unstimulated control group exhibited a 22% reduction in median seizure frequency, an effect equivalent to that obtained in the stimulated (treatment) group. This “control” effect persisted for at least three months [7]. An effect similar in magnitude and duration was reported by Morrell and colleagues in the non-stimulated condition from the RNS System in Epilepsy (NeuroPace) study [8].

Surgical procedures for seizure monitoring that involve only the placement of epidural grid electrodes, without penetrating brain tissue, have been anecdotally recognized to reduce the frequency of seizures post-operatively. These anecdotal observations are supported by the finding that after subdural grid electrode placement as a component of pre-surgical workup, more than 40% patients that did not undergo subsequent resection had Engel Class I, II, or III outcomes (i.e., a significant reduction in seizures) [9]. This raises the question of whether damage to the skull and/or the meninges may be sufficient to alter brain excitability, in the absence of implants into the brain parenchyma.

The effects of damage to the skull has recently been examined in the context of experimental models of traumatic brain injury, where craniotomy is commonly used as a surgical control (e.g., [10]). Cole and colleagues used a battery of behavioral tests to systematically compare naïve animals and those that underwent craniotomy [10]; they found that craniotomy was sufficient to cause significant impairment. In addition, up-regulated cortical cytokine levels have been reported after craniotomies [10], [11].

These findings, along with an incidental observation in our laboratory that animals that have undergone stereotaxic surgery involving craniotomy tend to show resistance to experimental seizures, led us to investigate whether damage to the skull in and of itself can alter seizure severity measured at various postoperative time points.

For our experiments, we placed bilateral craniotomies in the rat skull. Between 3 and 20 days postoperatively, animals were tested for behavioral seizure responses to a chemoconvulsant challenge (pentylenetetrazole, PTZ). We found that craniotomy significantly attenuated seizures evoked by PTZ 3, 6, and 10 days post-operatively.

## Materials and Methods

### Ethics statement

All procedures were performed in compliance with the American Association for Accreditation of Laboratory Animal Care (AAALAC) standards, and were approved by the Georgetown University Animal Care and Use Committee.

### Animals

Male, Sprague Dawley rats were purchased from Harlan Laboratories (Frederick, MD), and housed in the Division of Comparative Medicine at Georgetown University with food and water available ad libitum. Animals were pair housed in a temperature and humidity controlled room with a standard light-dark cycle (0600∶1800 Lights on). All experiments were conducted during the light cycle. Animals were between 220 and 300 g at the time of surgery.

### Treatments

Three groups of animals were used in these experiments. One group received bilateral craniotomies of 2.5 mm in diameter (n = 26), a second group received bilateral craniotomies of 3.5 mm in diameter (n = 28), and the third group (sham-operated controls, n = 40) underwent all surgical procedures except for craniotomies. Within these groups, the majority of animals (n = 47) were repeatedly tested for seizure responses using a 70 mg/kg challenge dose of PTZ at 3, 6, and 10 days postoperatively. A smaller group (n = 20) was tested using 70 mg/kg PTZ given once on day 20. Finally, the remaining 27 animals (15 shams, 12 2.5 mm craniotomies) were used to test different doses of PTZ at 3 days and 10 days postoperatively. The order of testing for these 27 animals was counterbalanced (as described in Table S1) to control for order effects. The numbers of animals used for statistical comparison are indicated in each figure.

### Surgical procedures

**Figure 1:**
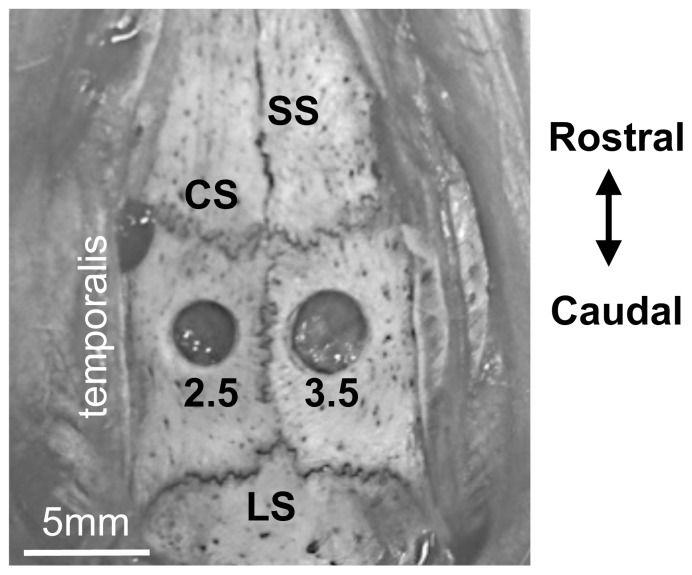
Position and size of craniotomies in the parietal bone of the rat skull. SS =  sagittal suture, CS =  coronal suture, LS =  lambdoidal suture. Scale bar indicates 5 mm. 2.5 and 3.5 indicate the diameter of the craniotomy.

Animals were anesthetized with Equithesin (a combination of sodium pentobarbital, chloral hydrate, magnesium sulfate, ethanol, and propylene glycol) (2.5 ml/kg, i.p.) and placed in a standard rat stereotaxic frame (Kopf). After shaving the hair on the surface of the skull, the skin was scrubbed with Betadine and alcohol before making a 2.5 cm incision at the midline. The surface of the skull was then cleaned with gauze. In pilot experiments (data not shown), craniotomies were made using a manual trephines and a high-speed (5,000–10,000 RPM) Dremel (Robert Bosch Tool Corporation) tool with a 2 mm rounded diamond burr. We chose the Dremel for making the craniotomies in the present experiments because it allowed us to have greater control over the size and reproducibility of the craniotomies. In pilot experiments, using either method of craniotomy placement, there was no visible damage to the dura or the underlying cortex when postmortem brains were visually inspected. Craniotomies were made in the parietal bone midway between the coronal and lambdoidal sutures in the anteroposterior plane and midway between the sagittal suture and the temporalis muscle in the mediolateral plane (Figure 1). Drilling was stopped as soon as the burr penetrated the depth of the skull, avoiding contact with the underlying dura mater. Gelfoam (Baxter Healthcare) moistened with sterile isotonic saline was placed on the dura to fill the hole and the skin was then sutured and dressed with topical antibiotic and lidocaine ointments. Control (sham-operated) animals underwent the same surgical procedures with the exception of the craniotomies.

### Seizure testing

Testing took place on postoperative days 3, 6, 10, and 20. Seizures were evoked using pentylenetetrazole (PTZ, Sigma-Aldrich; 50–90 mg/kg, s.c.). PTZ was selected as the method of seizure induction for this study because it induces seizures ranging from myoclonic jerks and automatisms to clonic/tonic manifestations. Additionally, we have previously tested animals repeatedly using PTZ and obtained stable responses with no signs of tolerance or sensitization [12]. A dose of 70 mg/kg was selected as our standard test dose because this dose causes moderately severe seizures (Figure S1). Seizures were scored using a point system for seizure severity. Individual manifestations were assigned points and summed for a maximum score of 12. The point values were as follows: myoclonic jerks = 0.5, facial+forelimb clonus = 1, facial+forelimb clonus with rearing (with or without loss of balance) = 1.5, clonic/tonic body twist = 2, running/bouncing clonus = 3, tonic forelimb extension = 4. The dose-dependency of this rating system is shown in Figure S1. Note that the seizure endpoints with these doses of PTZ are beyond the ceiling effect for discrimination by EEG analysis [13]. Latency to the onset of seizure activity was also recorded. Animals were observed for up to 60 min.

### Data analysis

Parametric data (seizure latencies) were analyzed using one-way analysis of variance (ANOVA) with Dunnett's post-hoc tests (one-tailed); these analyses were run within each time point. Seizure scores (non-parametric data) were analyzed using Kruskal-Wallis test followed by Dunn's post-hoc test; these analyses were run within each time point. For the time course data (3, 6, and 10 days post-operative), seizure scores were also analyzed using a two-way Aligned Rank Transformed (ART) ANOVA for non-parametric two-way designs (seizure score as a function of post-operative day and trephination status, [14]). 20-day data were not included in this analysis because only sham and 3.5mm trephine groups were tested at 20 days, yielding an unbalanced design for the ANOVA. Seizure latency for the time course data was also analyzed by two-way ANOVA (latency to seizure onset as a function of post-operative day and trephination status).

## Results

### Response to 70 mg/kg PTZ

In response to 70 mg/kg PTZ (s.c.), sham operated animals exhibited seizure responses with mean latencies for the control groups ranging from 329 to 401 sec across the various postoperative testing days. During the 60 min observation period, multiple seizure manifestations occurred, generally starting with myoclonic jerks followed by facial and forelimb clonus (with or without rearing), and progressing to clonic/tonic body twist by 10–20 min after injection. A subset of animals progressed to running/bouncing clonus with or without tonic forelimb extension. Mean scores for the sham-operated groups ranged from 5.0 to 7.4.

**Figure 2:**
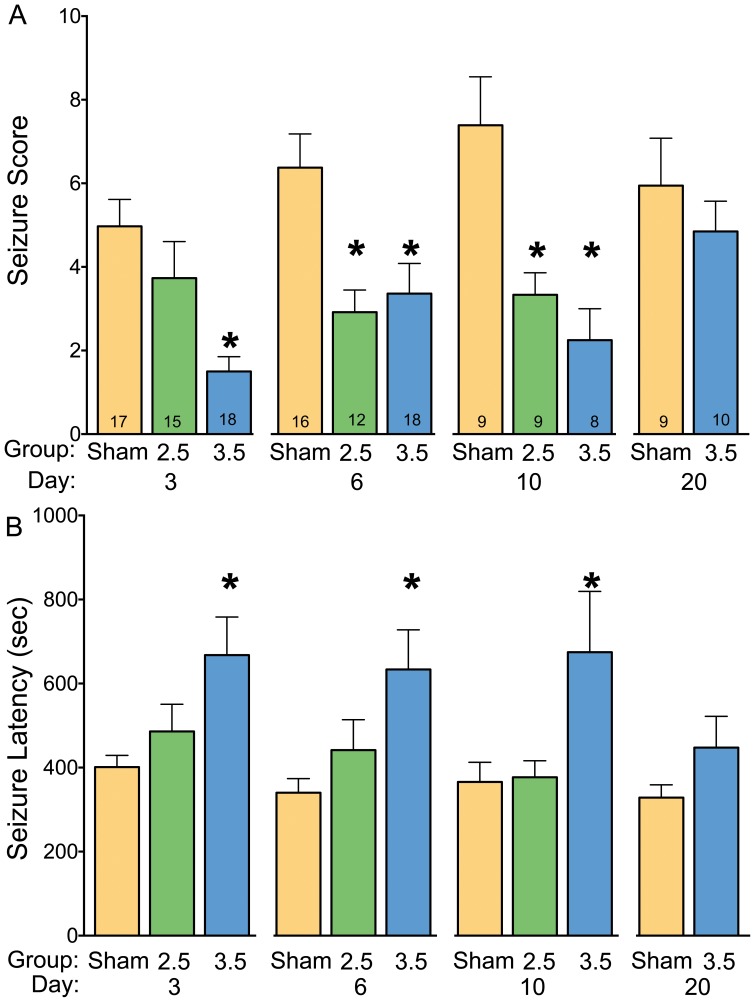
Effect of craniotomy on seizure score and latency to seizure onset. (A) Seizure score as a function of group (i.e., Sham controls, 2.5 mm and 3.5 mm craniotomies) and post-operative testing day (3, 6, 10, and 20 days post-surgery). The numbers on each bar indicate the number of animals in each group. *  =  seizure score was significantly reduced as compared to the equivalent control (sham) group (P<0.05, Dunn's post-hoc). (B) Latency to seizure onset (in seconds) as a function of group (as described above). *  =  latency is significantly greater than sham controls (P<0.05, Dunnett's multiple comparison test).

Three days postoperatively, animals with 3.5 mm burr holes showed both a significant reduction in seizure severity (P<0.0001, Fig 2A) and a significant increase in latency to seizure onset (P<0.05, Fig 2B) as compared to sham controls. Animals with 2.5 mm burr holes were not significantly different from sham controls with respect to either seizure severity or latency. These effects were revealed by Kruskal-Wallis test, which showed a main effect of group for seizure severity (H = 18.9, d.f., 49, P<0.0001), and ANOVA which revealed a main effect of group on seizure latency (F_2,46_ = 4.3, P<0.05).

Six days postoperatively, both large and small craniotomies were associated with a significant reduction in seizure severity (P<0.05 and P<0.01, respectively) as compared to sham controls (Fig 2A). An increase in latency to seizure onset as compared to sham-operated controls was observed for animals with 3.5 mm burr holes (P<0.01, Fig 2B). Ten days after surgery, an identical pattern of effects was seen: both the large and small craniotomies reduced seizure severity as compared to sham operated controls (P<0.05 and P<0.005, respectively), with the large craniotomies also showing an increase in seizure latency (P<0.05). For the animals with craniotomies that exhibited limbic motor seizures (facial+forelimb clonus with or without rearing), the majority (7/12) had only one episode during the one-hour observation period, whereas almost all controls (8/9) exhibited multiple seizures during the same period. This difference in seizure frequency was significant (Fisher's exact test, one-tailed, P<0.05). The period of time over which seizure activity occurred (measured from the first to last seizure manifestation) did not differ across treatment groups (F_2,24_ = 0.41, P = 0.67).

Seizure scores were analyzed using Kruskal-Wallis test, which showed a main effect of group on seizure severity at six days (H = 10.63, d.f., 45, P<0.005), and ten days (H = 10.7, d.f. = 25, P<0.005). ART-ANOVA showed a main effect of treatment (F_2,113_ = 21.89, p<0.001), a trend toward a main effect of post-operative day (F_2,113_ = 2.591, p = 0.08) and no treatment-by-day interaction (F_4,113_ = 1.07, p = 0.38).

For latency to seizure onset, ANOVA revealed a significant main effect of group on seizure latency at both six (F_2,44_ = 4.5, P<0.05) and ten (F_2,25_ = 4.07, P<0.05) days postoperative. Two-way ANOVA revealed a main effect of treatment (F_2,111_ = 11.74, p<0.001), but neither a main effect of post-operative day (F_2,111_ = 0.42, p = 0.66), nor a treatment-by-day interaction (F_2,111_ = 0.16, p = 0.96).

When tested at 20 days postoperatively, animals with 3.5 mm craniotomies did not differ from controls with respect to seizure severity (U = 38.0, d.f. = 18, P = 0.78, Fig 2A) or seizure latency (t = 1.42, df = 17, p = 0.17, Fig 2B).

### Effect of craniotomy as a function of PTZ dose

**Figure 3:**
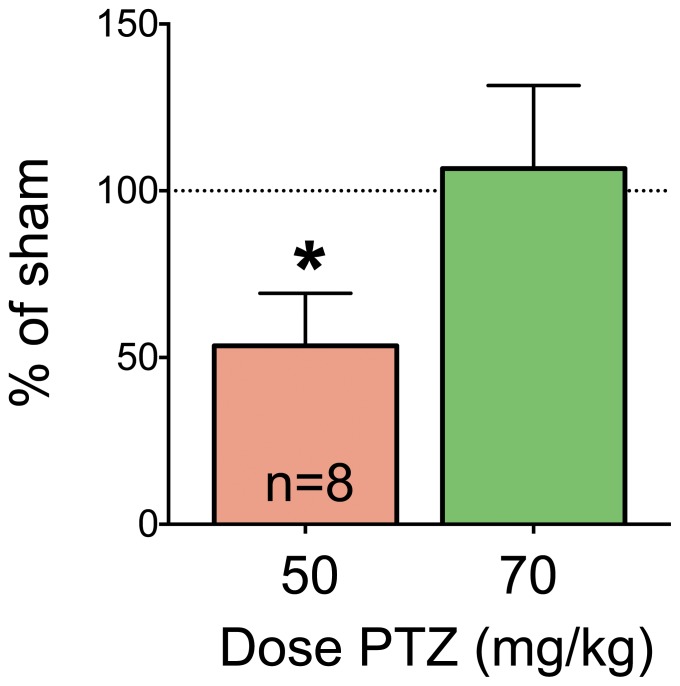
2.5/kg PTZ at 3 days post-operatively. Data are expressed as % of respective shams for animals with 2.5 mm craniotomies challenged with 50 or 70 mg/kg of PTZ. We normalized each treated group to the median of the matched sham control group (a value of 100% equals sham control response). The data shown for 70 mg/kg represent a transformation of the data shown in Figure 2 for comparison purposes. Sham animals challenged with 50 mg/kg PTZ displayed a mean seizure score of 4.6 (median  = 3.5); those challenged with 70 mg/kg displayed a mean of 5.0 (median  = 3.5). Animals with craniotomies displayed means of 1.9 (median  = 2.25) and 3.7 (median  = 2.5) for 50 and 70 mg/kg challenge doses, respectively. * indicates significantly different than 100% (Wilcoxon Sign Rank test, P<0.05).

To determine if the effect of craniotomy varied with dose of PTZ challenge, we examined the effect of 2.5 mm craniotomies at 3 and 10 days postoperatively using two different doses of PTZ at each time point. Because we observed no protection against 70 mg/kg PTZ at 3 days, we tested a lower dose (50 mg/kg) at this time point. At 10 days, we tested a higher dose (90 mg/kg) because we previously found significant protection against 70 mg/kg. On postoperative day 3, sham control animals challenged with 50 mg/kg PTZ displayed seizures characterized by myoclonic jerks and facial+forelimb clonus (with rearing). Animals with 2.5 mm burr holes had significantly less severe seizures than did sham controls in response to this dose of PTZ (P<0.05, median test, Fig 3A).

**Figure 4:**
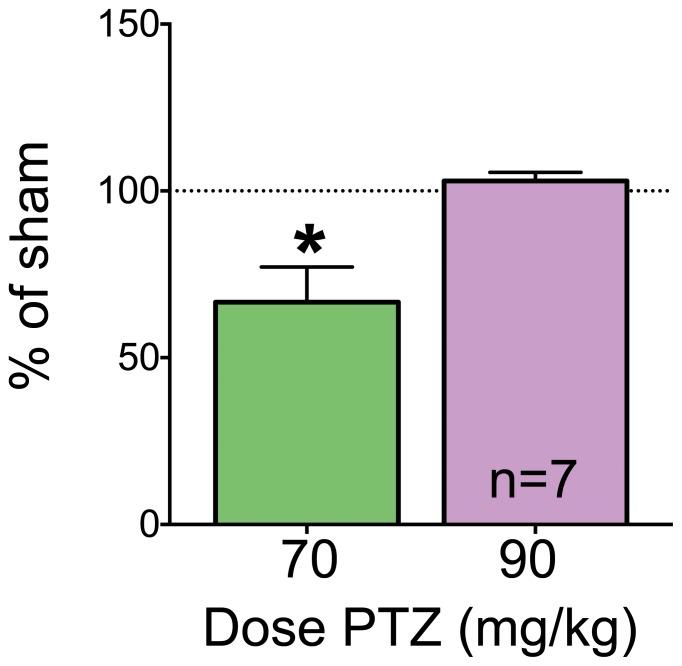
2.5/kg PTZ at 10 days post-operatively. Data are expressed as % of respective shams for animals with 2.5 mm craniotomies challenged with 70 or 90 mg/kg of PTZ. We normalized each treated group to the median of the matched sham control group (a value of 100% equals sham control response). The data shown for 70 mg/kg represent a transformation of the data shown in Fig 2 for comparison purposes. Sham animals challenged with 90 mg/kg PTZ displayed a mean seizure score of 10.5 (median  = 10.5). Animals with craniotomies displayed means of 3.3 (median  = 3.5) and 11.57 (median  = 12) for 70 and 90 mg/kg challenge doses, respectively. * indicates significantly different than 100% (Wilcoxon Sign Rank test, P<0.05).

When a high dose of PTZ (90 mg/kg) was given to animals with 2.5 mm burr holes on post operative day 10, the seizure response did not differ from that in sham controls (P>0.05, Wilcoxon Sign Rank Test, Fig 4). The seizures in both groups were characterized by severe seizures including clonic and tonic manifestations.

## Discussion

Here we have shown that craniotomies significantly reduce seizure severity in rats. This effect lasts for at least 10 days postoperatively, with a return to control levels of seizure response by 20 days. By comparing craniotomies of two diameters, 2.5 mm and 3.5 mm, we found that the effect was size-dependent. Our data demonstrate that damage to the skull without direct brain injury can have a significant impact on post-surgical neurological status and support the value of appropriate surgical controls in both basic animal models of CNS disorders and in clinical neurosurgical trials.

These findings extend those of Cole and colleagues [10], who found that craniotomy alone was sufficient to alter selective behaviors (startle responses), during the two weeks following craniotomy. While these authors discussed the implications of their findings for animal models of traumatic brain injury, our results suggest the implications extend to animal models of epilepsy, and potentially other CNS disorders.

From the perspective of preclinical epilepsy research, our data suggest that the penetration of the skull required for placing electrodes, cannulae, or lesions, may in and of itself alter networks responsible for seizure generation. This confound may be minimized by allowing a sufficient period of postoperative recovery before initiating measurements after brain lesions or implants. Our present findings may shed light on an effect previously described during amygdala kindling in rats [15]–[17]. These studies reported a higher afterdischarge threshold (ADT) and slower kindling in animals in which stimulation was initiated one week postoperatively, as compared to animals in which stimulation was initiated after a delay (one month postoperatively). The authors suggested that the electrodes alone might have had a pro-kindling effect that required several weeks to develop. The craniotomy effect that we describe suggests an alternative explanation: the elevated ADT seen in the early postoperative period may reflect the craniotomy effect, which abates by three weeks postoperatively. These functional changes, coupled with craniotomy-induced biochemical changes, including increases in cytokine levels [10], [11] and altered gene expression [18], [19], reinforce the notion that penetration of the skull is not neurologically inert, and should be controlled for in both preclinical and clinical neurosurgical investigations.

Clinically, the reduction in seizures that has been reported after implantation of recording electrodes [5] or after the implantation of stimulating electrodes (but before the onset of stimulation) [6]–[8] has proved an impediment for interpreting clinical trial results. This implant effect has provoked speculation regarding mechanisms, including the possibilities of electrode-induced inflammation and/or microlesions [20], [21]. Our data raise the possibility that damage to the skull may be sufficient to account for some of these clinical observations. This possibility is consistent with the clinical observations that the implantation of epidural grid electrodes that do not penetrate brain tissue transiently alters the pattern and/or frequency of seizures during the weeks following implantation.

These findings provide a foundation for a more detailed assessment of the impact of craniotomy, including the degree to which craniotomy may exert anticonvulsant effects in other models of epilepsy. Additionally, it is possible that a lower dose of PTZ may be sensitive to the craniotomy effect at 20 days (once protection is no longer evident in response to 70 mg/kg). Finally, the degree to which changes in cytokine levels (as described above) or in other factors induced by craniotomy (e.g., bone morphogenetic proteins, fibroblast growth factors; [22]) predict or contribute to the anticonvulsant effect of craniotomy can now be explored.

It would also be worthwhile to examine how craniotomies impact the pattern of seizure initiation and propagation through various cortical and subcortical networks. Because implantation of either epidural or depth electrodes requires drilling holes through the skull, EEG electrodes cannot be used for this purpose. Instead, non-invasive imaging techniques or postmortem immunohistochemistry may be of great value for such an investigation.

The relevance of our findings may extend beyond neurosurgical treatments for epilepsy. In clinical trials for Parkinson's disease therapies involving neuro-transplantation [23]–[26], gene transfer, or DBS, the importance of surgical controls has gained widespread attention largely due to mounting evidence for therapeutic actions of surgical control procedures [20], [27]–[29].

It is tempting to speculate that our findings may provide an explanation for a procedure that has been used to treat disorders such as epilepsy for over 7,000 years: trepanation [30]. According to the archeological record, this practice was employed in both the Old and New Worlds. Trepanned skulls have been found from Alsace [30] to the Andes [31], with the procedure recommended by Hippocrates [32] and practiced by surgeons in ancient Rome, Greece, and China [33]–[35]. In some cases, the procedure was done repeatedly, with multiple holes at various stages of healing found in single skulls [36], [37]. In fact, trepanation was performed during the Renaissance as a treatment for epilepsy, in some cases to “remove evil air” [38], [39]. While the explanations at the time were largely based on superstition, our present findings raise the possibility that the application of trepanation may have been reinforced by therapeutic outcomes. This could account for the widespread nature of this practice in the treatment of seizures across cultures and millennia.

## Conclusions

The anticonvulsant effect we described following the placement of craniotomies demonstrates that damage to the skull cannot be considered an inert intervention and that it thus represents a potential confound in preclinical and clinical neurosurgical evaluation. These data add to the existing evidence arguing for appropriate neurosurgical controls.

## Supporting Information

Figure S1 **Dose-dependency of the seizure rating scale.** Seizure score increases significantly with increasing dose of PTZ (Kruskal-Wallis test, H = 31.1, d.f., 78, P<0.0001). * P<0.05, ** P<0.001 (Dunn's post-hoc test, one-tailed). 6 animals (4 treated with a 50 mg/kg dose of PTZ, and 2 treated with a 90 mg/kg dose of PTZ were excluded as outliers based on Tukey Boxplots).
(TIF)

Table S1 **Schedule of testing showing the number of animals tested on each experimental schedule.**
(TIF)
