# Supplementary figures and images for: Delayed Effect of Craniotomy on Experimental Seizures in Rats
Source: PLoS One. 2013 Dec 4;8(12):e81401. doi: 10.1371/journal.pone.0081401 (PMC3852486; doi:10.1371/journal.pone.0081401)

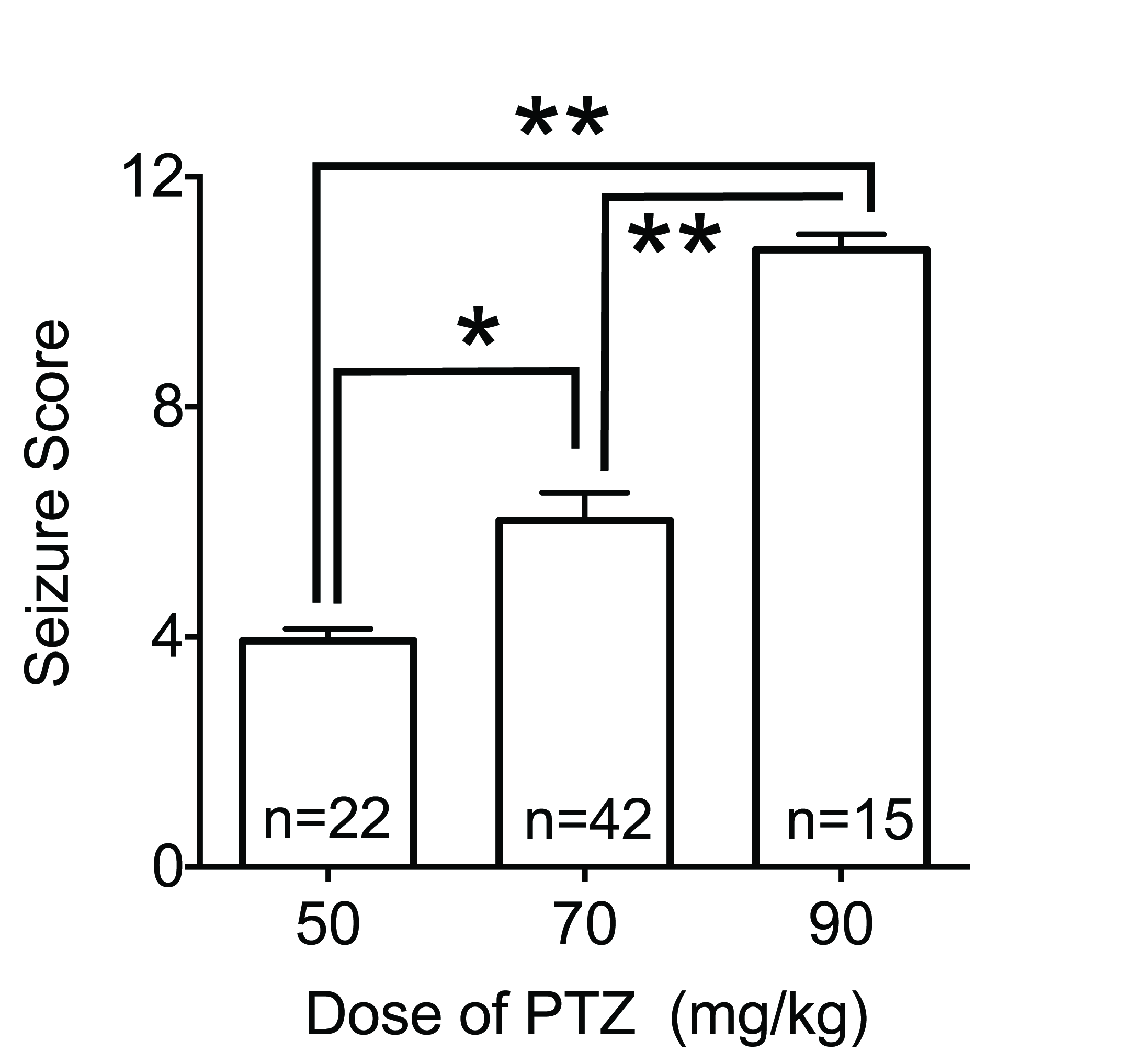

Supplement: Figure S1 — Dose-dependency of the seizure rating scale. Seizure score increases significantly with increasing dose of PTZ (Kruskal-Wallis test, H = 31.1, d.f., 78, P<0.0001). * P<0.05, ** P<0.001 (Dunn's post-hoc test, one-tailed). 6 animals (4 treated with a 50 mg/kg dose of PTZ, and 2 treated with a 90 mg/kg dose of PTZ were excluded as outliers based on Tukey Boxplots). (TIF) [file pone.0081401.s001.tif]

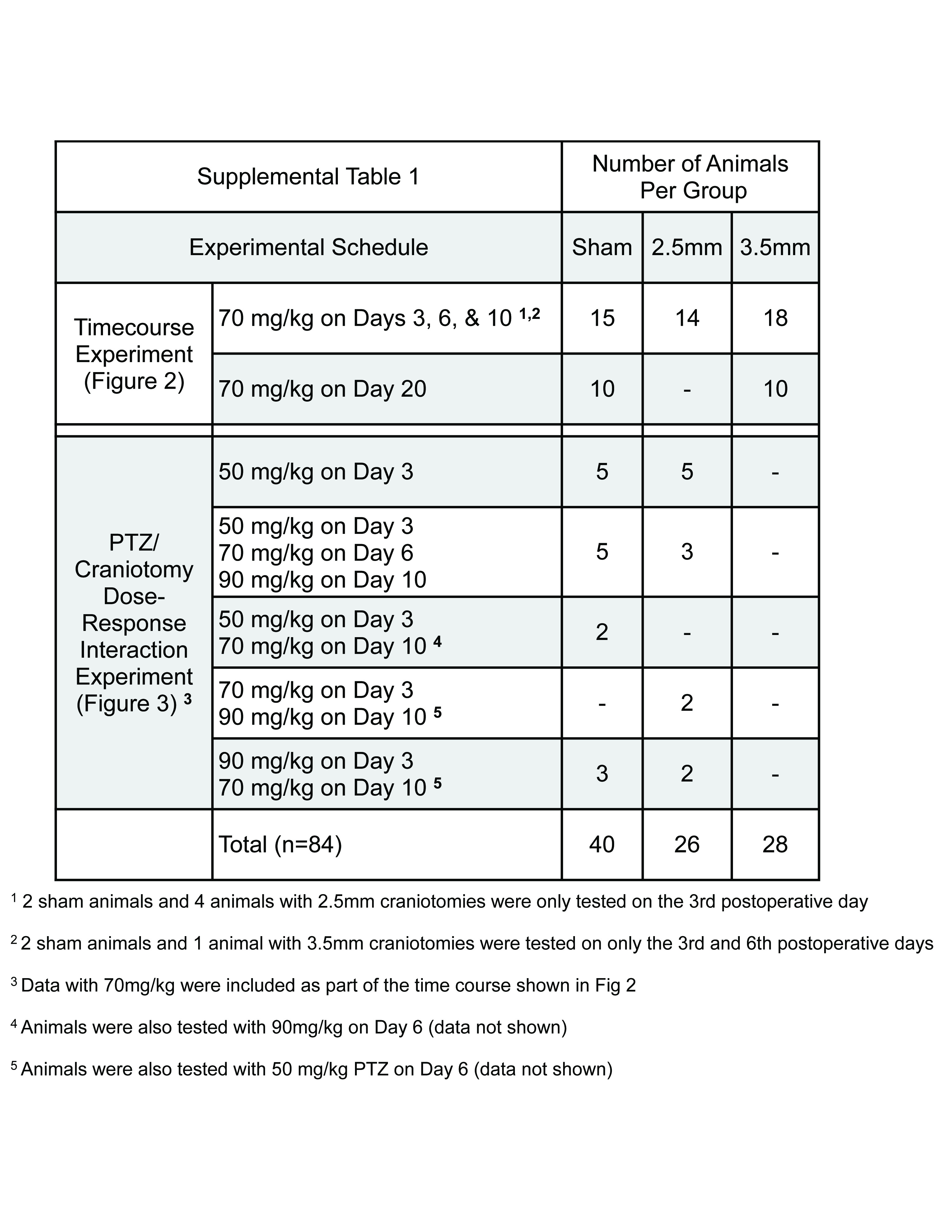

Supplement: Table S1 — Schedule of testing showing the number of animals tested on each experimental schedule. (TIF) [file pone.0081401.s002.tif]
